# De novo biosynthesis of *p*-coumaric acid and caffeic acid from carboxymethyl-cellulose by microbial co-culture strategy

**DOI:** 10.1186/s12934-022-01805-5

**Published:** 2022-05-10

**Authors:** Miao Cai, Jiayu Liu, Xiaofei Song, Hang Qi, Yuanzi Li, Zhenzhou Wu, Haijin Xu, Mingqiang Qiao

**Affiliations:** 1 The Key Laboratory of Molecular Microbiology and Technology, Ministry of Education, College of Life Sciences, Nankai University, Tianjin, 300071 China; 2 College Biotechnology and Bioengineering, Zhejiang University of Technology, Hangzhou, 310014 China; 3 School of Light Industry, Beijing Technology and Business University (BTBU), Beijing, 100048 China

**Keywords:** *Saccharomyces cerevisiae*, Co-culture, Carboxymethyl-cellulose, *p*-coumaric acid, Caffeic acid

## Abstract

**Background:**

Aromatic compounds, such as *p*-coumaric acid (*p*-CA) and caffeic acid, are secondary metabolites of various plants, and are widely used in diet and industry for their biological activities. In addition to expensive and unsustainable methods of plant extraction and chemical synthesis, the strategy for heterologous synthesis of aromatic compounds in microorganisms has received much attention. As the most abundant renewable resource in the world, lignocellulose is an economical and environmentally friendly alternative to edible, high-cost carbon sources such as glucose.

**Results:**

In the present study, carboxymethyl-cellulose (CMC) was utilized as the sole carbon source, and a metabolically engineered *Saccharomyces cerevisiae* strain SK10-3 was co-cultured with other recombinant *S. cerevisiae* strains to achieve the bioconversion of value-added products from CMC. By optimizing the inoculation ratio, interval time, and carbon source content, the final titer of *p*-CA in 30 g/L CMC medium was increased to 71.71 mg/L, which was 155.9-fold higher than that achieved in mono-culture. The de novo biosynthesis of caffeic acid in the CMC medium was also achieved through a three-strain co-cultivation. Caffeic acid production was up to 16.91 mg/L after optimizing the inoculation ratio of these strains.

**Conclusion:**

De novo biosynthesis of *p*-CA and caffeic acid from lignocellulose through a co-cultivation strategy was achieved for the first time. This study provides favorable support for the biosynthesis of more high value-added products from economical substrates. In addition, the multi-strain co-culture strategy can effectively improve the final titer of the target products, which has high application potential in the field of industrial production.

**Supplementary Information:**

The online version contains supplementary material available at 10.1186/s12934-022-01805-5.

## Background

*p*-Coumaric acid (*p*-CA), a secondary metabolite in plants and mushrooms, is bioconverted from tyrosine or phenylalanine, and it has various biological activities such as antioxidant, anti-inflammatory, and anticancer activities [1–3]. *p*-CA is also an important precursor of phenolic acids [4], flavonoids [5], and stilbenes [6]. Caffeic acid is derived from *p*-CA and shows stronger pharmacological activities [7–9], and its derivatives chlorogenic acid [10], rosmarinic acid [11], and caffeic acid phenethyl ester [12] also have important medicinal values. Both *p*-CA and caffeic acid have been widely used in pharmaceutical, food, and cosmetic industries [13, 14]. During the production of chemicals, the use of fossil energy and organic reagents has irreversible effects on the environment. The long culture period of plants and the complex extraction procedures also limit the production and application of such compounds [15]. To meet the market demand, researchers have focused their attention on heterologous biosynthesis of aromatic compounds in microorganisms by using synthetic biology and metabolic engineering systems [16–21]. In our previous studies, engineered strains of *Saccharomyces cerevisiae* were used as host cells to improve the yield of aromatic amino acids and produce multiaromatic compounds [22, 23].

To date, numerous studies have been conducted on the heterologous synthesis of aromatic compounds, and most of these studies utilized glucose as the sole or main carbon source. Unlike starch materials, which are costly and occupy limited agricultural land, lignocellulose is a renewable non-food agricultural resource and abundant in the world [24, 25]. However, because of high polymer biomass, the biodegradation of lignocellulose is restricted unless it is pretreated to reduce cellulose crystallinity and lignin content. With advances in metabolic engineering, enzyme engineering, and synthetic biology, several microbial strains have been designed and recombined to possess or enhance the capability of cellulose degradation and further yield industrial products [26–28], especially for biofuels. In our previous laboratory studies, we constructed a series of cellulase-expressing yeast strains through a *POT1*-mediated *δ*-integration strategy to yield bioethanol in a cellulose-based medium [29].

The complex processes of cellulase expression, cellulose decomposition, and fermentation of high value-added products will increase the metabolic burden of a single strain. To balance the pathways, a co-culture system was used in this study to achieve the bioconversion of carboxymethyl-cellulose (CMC) to aromatic compounds. The technique of culturing two or more cell populations simultaneously is termed as co-culture. Unlike natural mixed culture, co-culture technology is used to study interactions between species, generate new products, or improve yield through a purposeful and conscious use of high-throughput technology and a bioinformatics platform [30–32]. In recent years, co-cultivation technology has often been applied to the study of high-efficiency transformation of un-conventional biomass, such as lignocellulose. The production of methyl halide from untreated cellulosic materials was achieved by co-cultivating an engineered yeast strain and cellulolytic bacterium *Actinotalea fermentans* [33]. Nakayama et al. co-cultured the cellulolytic bacterium *Clostridium thermocellum* with the butanol-producing strain *Clostridium saccharoperbutylacetonicum* to efficiently convert crystalline cellulose to butanol [34]. A *Trichoderma reesei*–*Escherichia coli* co-culture system was also used to produce isobutanol from microcrystalline cellulose and pretreated corn stover [35].

In the present study, a co-culture system was constructed to investigate the potential of conversion from CMC to aromatic compounds. The CMC-degrading *S. cerevisiae* strain SK10-3 [36] and an engineered *S. cerevisiae* strain NK-B2 [22], which is a high tyrosine-producing strain, were used. Various parameters that may affect the operation of the co-cultivation system, including inoculation ratio, interval time, and carbon source content, were adjusted to optimize the final yield.

## Results

### Co-culture system construction

To achieve the biosynthesis of high value-added products from lignocellulose, an efficient CMC-degrading strain, SK10-3, was chosen for further engineering. The tyrosine ammonia lyase-encoding gene from *Rhodobacter capsulatus* was introduced into SK10-3 for *p*-CA synthesis. The de novo biosynthesis of *p*-CA from CMC was achieved; however, the degrading pathway of CMC in SK10-3 increased its metabolic burden, with only 0.46 mg/L *p*-CA detected in 10 g/L CMC medium. Even in the 20 g/L glucose medium, the yield was only 2.14 mg/L *p*-CA, which was much lower than that of the control strain BY4741b (4.98 mg/L).

**Fig. 1:**
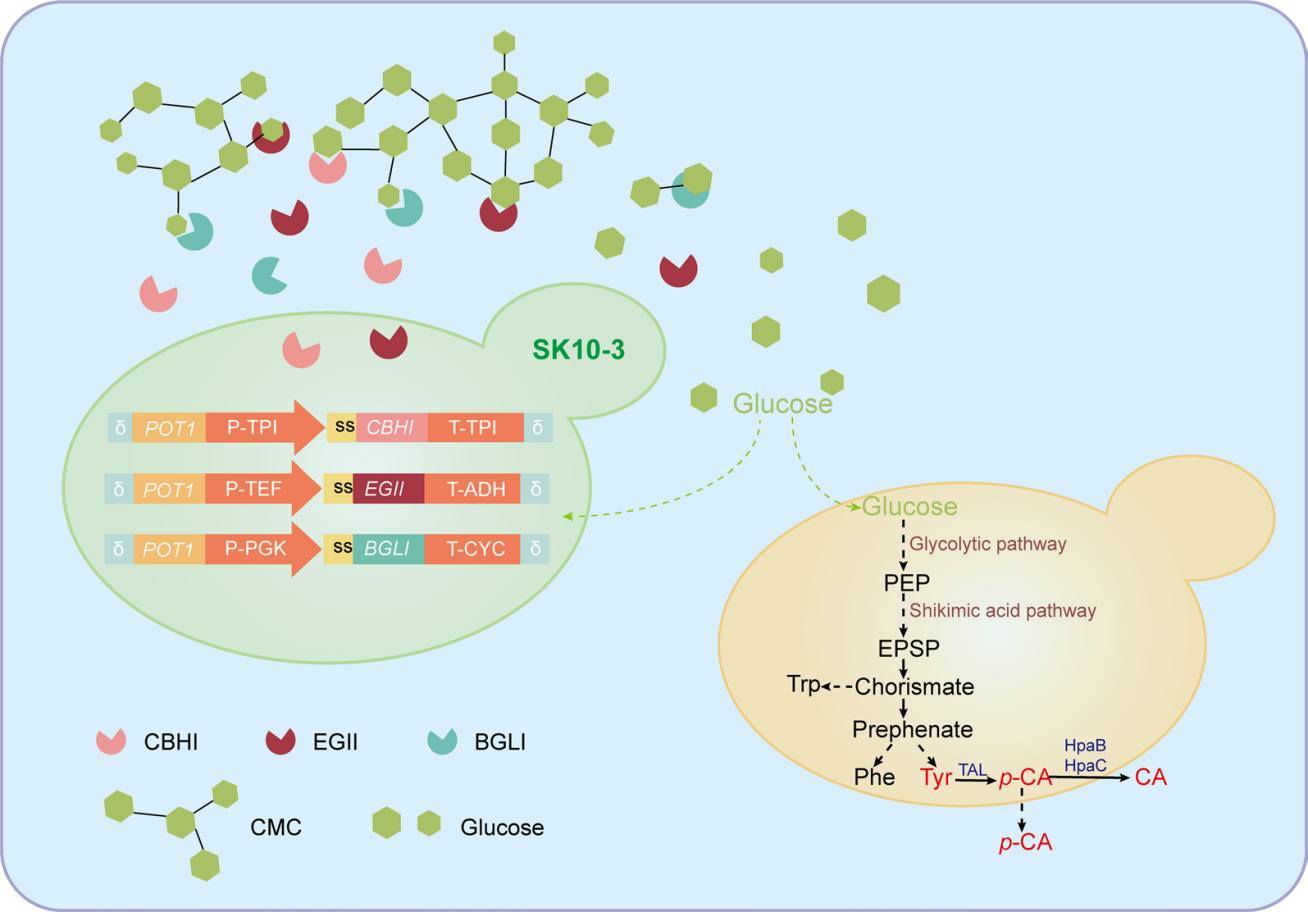
Schematic illustration of the co-culture system of different metabolically engineered *S. cerevisiae* strains for de novo biosynthesis of *p*-CA and CA from CMC. In this system, CMC in medium was degraded by cellulolytic enzymes expressed in SK10-3 by the *POT1*-mediated *δ*-integration strategy. The released glucose was assimilated by SK10-3 and other co-culture strains to yield *p*-CA or CA. PEP: phosphoenolpyruvate; EPSP: 5-enolpyruvylshikimate-3-phosphate; Trp: tryptophan; Phe: phenylalanine; Tyr: tyrosine; *p*-CA: *p*-coumaric acid; CA: caffeic acid

**Fig. 2:**
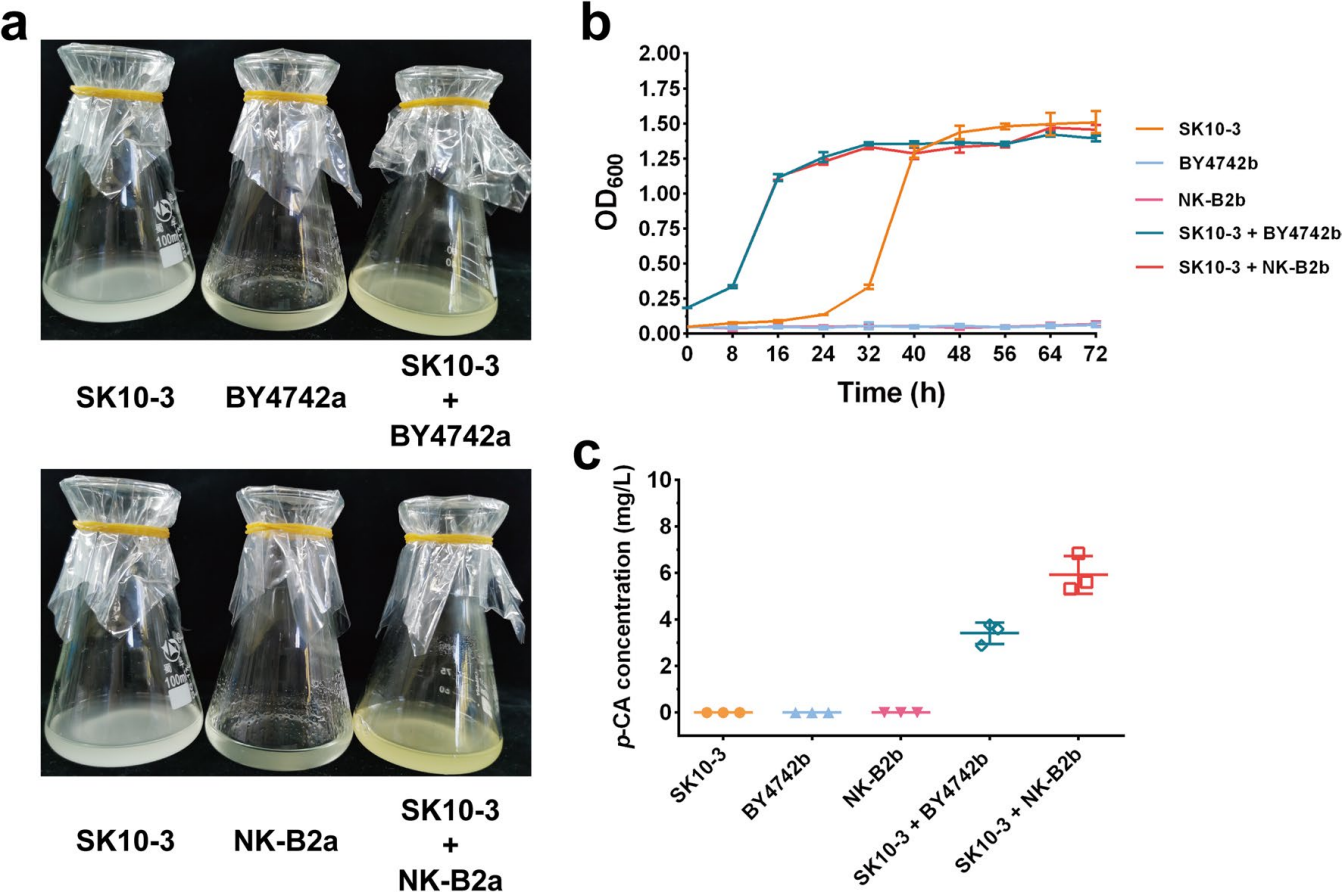
Phenotypes of betaxanthin and *p*-CA production in the co-culture systems in CMC medium. **a** Comparison of color phenotypes in mono-culture and co-culture systems. **b** Growth curves. **c** *p*-CA production in mono-culture and co-culture systems. Three replicates of each sample were used

The co-culture strategy was used to alleviate the metabolic stress of a single strain, and the high tyrosine-producing *S. cerevisiae* NK-B2 strain was selected for co-cultivation with SK10-3 and to ferment aromatic compounds. In this co-culture system, with CMC as the sole carbon source, the strain SK10-3 secretes cellulases to decompose CMC and provides monosaccharide glucose to the member strains for the biosynthesis of other high value-added compounds, as shown in Fig. 1. To verify the feasibility of this strategy, BY4742a and NK-B2a with synthases of betaxanthin were first co-cultured with SK10-3, and the color phenotype during fermentation was observed. Considering that the degradation of CMC in the medium requires a certain amount of time, a sequential co-culture strategy was adopted. SK10-3 was first inoculated into the 10 g/L CMC medium for 24 h, followed by inoculation of NK-B2a in the ratio of 1:1. After co-incubation of both strains for 48 h, the fermentation broth turned yellow (Fig. 2a), which indicated that BY4742a and NK-B2a could survive.

Subsequently, the NK-B2b strain, which could synthesize *p*-CA, was co-cultured with SK10-3, and the biomass and *p*-CA production in the system were measured (Fig. 2b). Although the growth of these strains was limited in the CMC medium, the synthesis of *p*-CA could still be detected (Fig. 2c). A total of 5.93 mg/L *p*-CA was accumulated in the SK10-3/NK-B2b co-culture system, and no *p*-CA was synthesized in SK10-3 mono-cultivation.

### Effect of the inoculum ratio and interval time on *p*-CA production

Because the cellulose saccharification efficiency of SK10-3 is influenced by its inoculation amount and incubation time, we monitored the changes in glucose content when SK10-3 was incubated with different inoculum doses. As shown in Table 1, the higher the inoculum dose of SK10-3, the faster is CMC degradation. CMC in the medium was almost completely saccharified after 36 h of incubation, indicating that there would not be enough carbon source in the system for NK-B2b growth if the interval time was longer than 36 h.

**Table 1:** Glucose content during SK10-3 mono-culture in 10 g/L CMC medium with different inoculum doses

Inoculation amount of SK10-3 (OD_600_)	Glucose content (mg/L) during mono-cultivation				
	0 h	12 h	24 h	36 h	48 h
0.075	–	31.98 ± 1.28	6.48 ± 2.04	–	–
0.067	–	29.10 ± 2.40	22.32 ± 1.54	4.50 ± 1.44	–
0.050	–	25.38 ± 1.43	25.56 ± 1.88	2.46 ± 0.91	1.38 ± 0.85
0.033	–	15.54 ± 0.75	26.40 ± 0.81	3.36 ± 1.33	3.72 ± 0.81
0.025	–	13.14 ± 1.18	29.10 ± 2.15	10.02 ± 1.26	4.11 ± 0.75

**–**: Indicates no glucose was detected

**Fig. 3:**
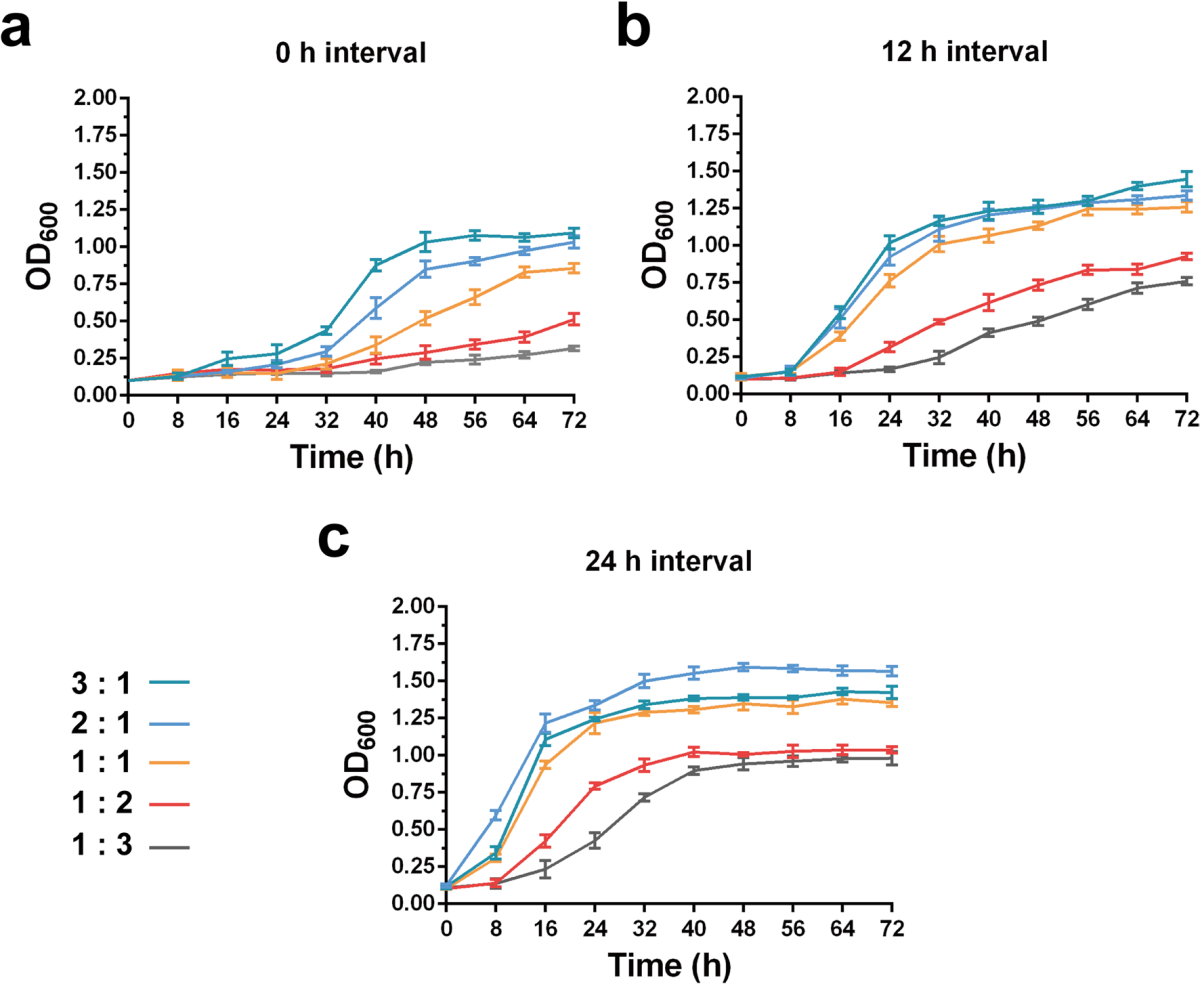
Growth curves of co-culture systems carried out according to different inoculum ratios of SK10-3 to NK-B2b and inoculation at different interval times. Three replicates of each sample were used

**Fig. 4:**
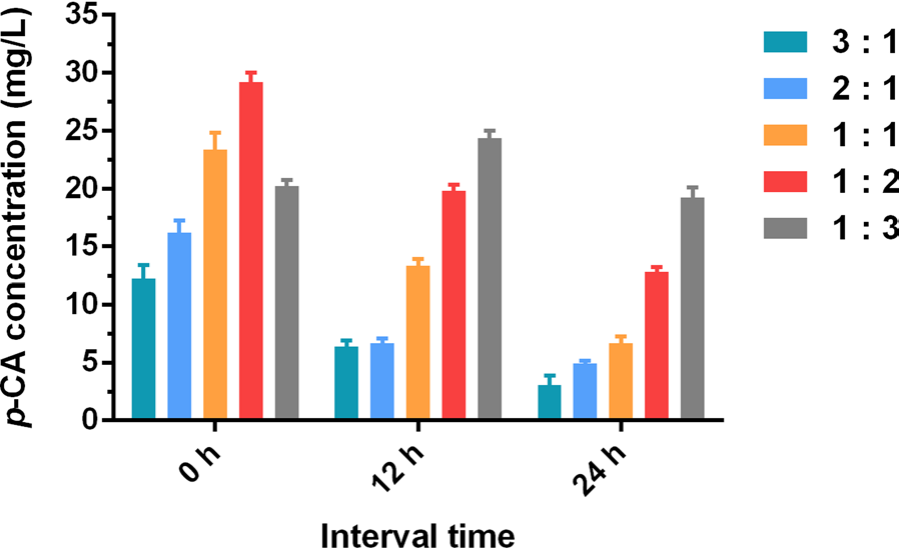
The *p*-CA production of co-culture systems carried out according to different inoculum ratios of SK10-3 to NK-B2b and inoculation at different interval times. Three replicates of each sample were used

The inoculum ratio is also an important factor to maintain the balance between bacterial growth and product yield in the co-culture system. Therefore, various ratios of SK10-3 to NK-B2b (3:1, 2:1, 1:1, 1:2, and 1:3) and interval times (0, 12, and 24 h) were investigated simultaneously. The total inoculum OD_600_ of the two engineered strains was 0.1. During the co-cultivation period, the growth was recorded and the production of *p*-CA was detected by HPLC after 120 h of co-culture fermentation (Figs. 3 and 4). As SK10-3 as the monosaccharide carbon source donor strain in the co-culture system, its inoculation sequence and ratio are important influencing parameters. Surprisingly, although the biomass was lowest when SK10-3 and NK-B2b were simultaneously inoculated, the highest *p*-CA titer (29.2 mg/L) was observed when these two strains were inoculated with the ratio of 1:2. Additionally, when the ratio of SK10-3 to NK-B2b inoculated simultaneously was 1:1 or the ratio was 1:3 and the interval time was 12 h, the production of *p*-CA was also considerable (23.4 and 24.3 mg/L, respectively). The glucose content of the co-culture systems was also monitored (Additional file 1: Table S1).

### Optimization of the total supply of the carbon source

**Fig. 5:**
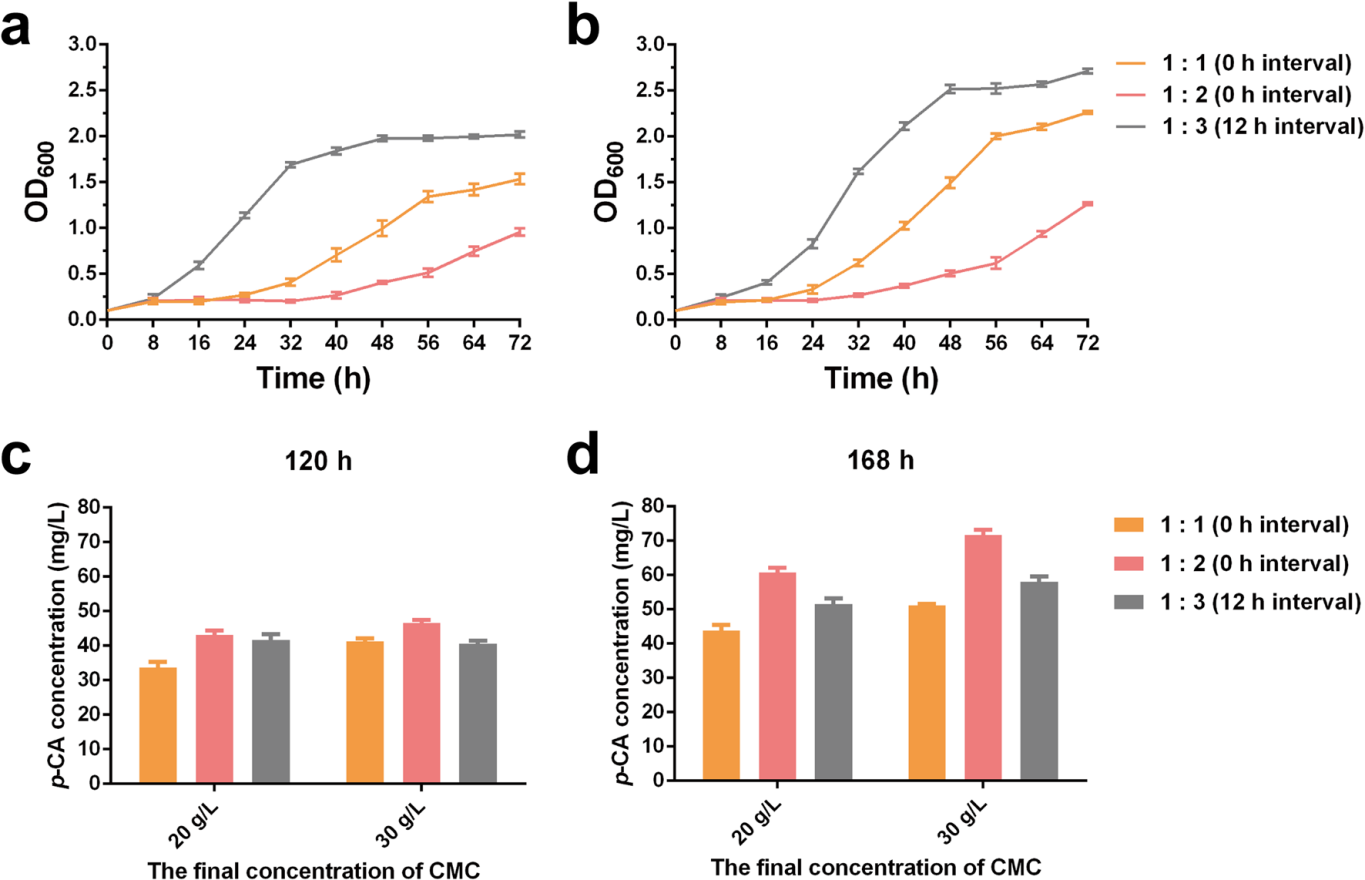
Growth curves and *p*-CA production in the carbon source optimization experiment. **a** Growth curves in 20 g/L CMC medium. **b** Growth curves in 30 g/L CMC medium. **c** *p*-CA production after 120 h fermentation. **d** *p*-CA production after 168 h fermentation. Three replicates of each sample were used

To further increase the production of *p*-CA in the co-cultivation system, the CMC content in the medium was improved. We increased the final concentration of CMC to 20 and 30 g/L, as adding too much CMC would make the medium thick and almost gel-like, which was not conducive to medium preparation and fermentation. Subsequently, the three optimal conditions from the previous result (Fig. 4) were selected to conduct the co-cultivation experiment in the high-carbon source medium, i.e., SK10-3 and NK-B2b inoculated simultaneously at the ratio of 1:1 or 1:2 or inoculated at an interval time of 12 h at the ratio of 1:3. The growth of these strains and *p*-CA titer were measured simultaneously (Fig. 5).

The results were consistent with the expectation: the higher the CMC concentration in the medium, the greater is the total biomass of the strain and the more is the amount of *p*-CA synthesized. After 120 h of co-cultivation, the production of *p*-CA was up to 46.55 mg/L in 30 g/L CMC medium (Fig. 5c), although this group had the lowest level of growth. This might be due to the medium with higher CMC content being denser and the contact between strains and cellulose was limited in the shaking culture process, thus hindering the efficiency of SK10-3 to degrade CMC. After 96 h of fermentation, high concentrations of glucose were detected in these co-culture samples, and CMC was almost completely decomposed and used after 168 h of co-cultivation (Additional file 1: Table S2). It is indicated that during the fermentation period of 120 h to 168 h in the high carbon source medium, the glucose content in the co-culture system could still meet the growth and metabolic synthesis of the strain. The *p*-CA titer after 168 h of fermentation was also measured, and the results were very optimistic. The highest *p*-CA titer was detected in the sample of SK10-3 and NK-B2b simultaneously inoculated at the ratio of 1:2, which was approximately 71.71 mg/L. Compared to the yield of 120 h, it was increased by 54% (Fig. 5d). Moreover, to determine the ratio of these two strains after co-culture fermentation, the spotting plate experiment was performed because NK-B2b cannot survive in CMC medium by mono-culture. In the optimum co-culture condition, the proportion of NK-B2b was 62% after fermentation.

### De novo biosynthesis of caffeic acid from CMC

**Fig. 6:**
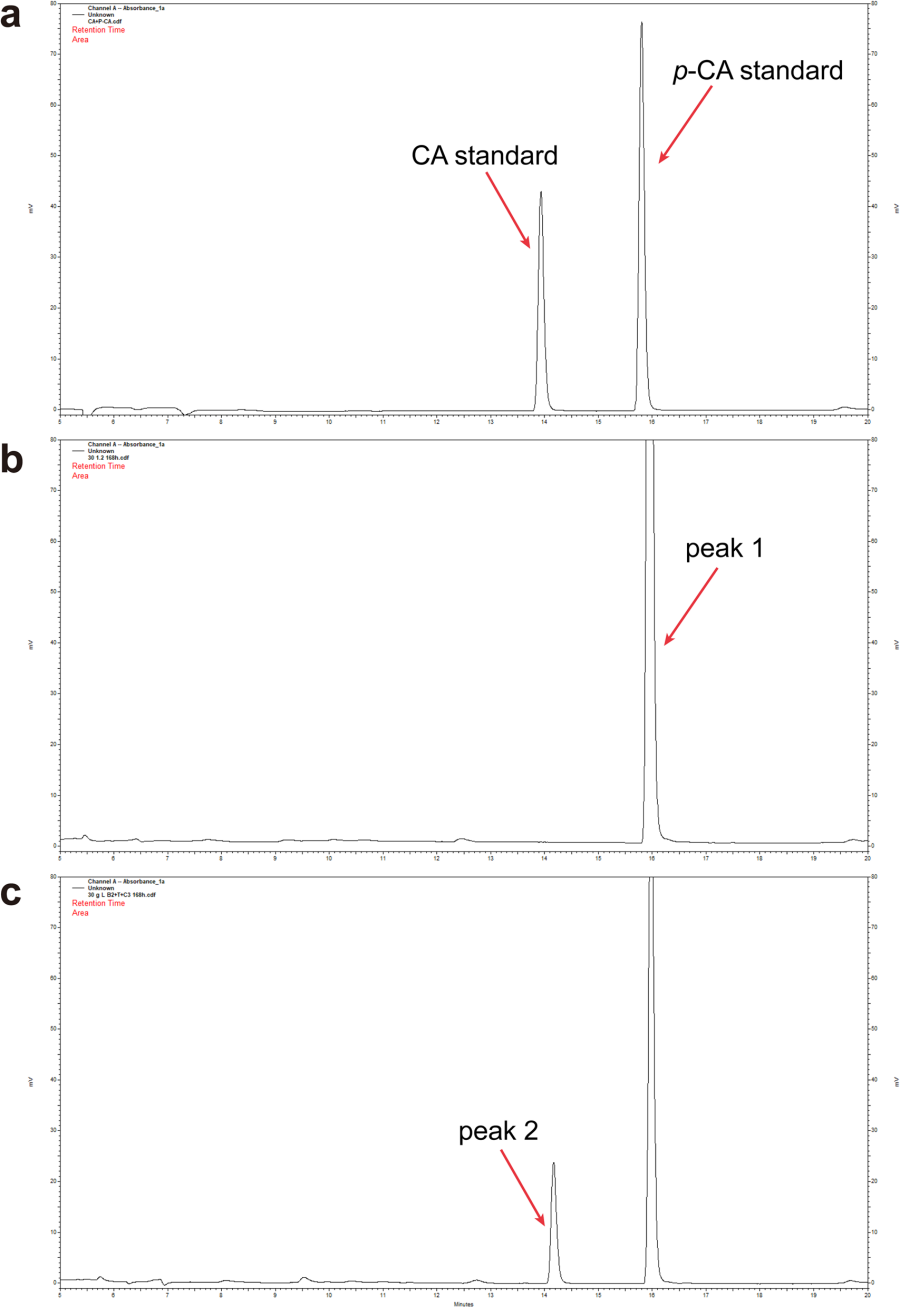
HPLC chromatogram of *p*-CA and caffeic acid. **a** Standards of *p*-CA and caffeic acid. **b** Co-culture sample of SK10-3 and NK-B2b; peak 1 was *p*-CA obtained from this co-culture system. **c** Co-culture sample of SK10-3 and NK-B2c; peak 2 was caffeic acid obtained from this co-culture system

A derivative compound of *p*-CA, caffeic acid, was attempted to biosynthesize from CMC in the co-culture system. Based on the NK-B2b strain, the caffeic acid-synthesizing strain NK-B2c was constructed by transforming the codon-optimized caffeic acid synthase genes *hpaB* of *Pseudomonas aeruginosa* and *hpaC* of *Salmonella enterica* [37, 38]. SK10-3 was co-cultured with NK-B2c under the optimal condition we screened before. After 168 h of fermentation, 8.33 mg/L caffeic acid was detected by HPLC (Fig. 6) from 30 g/L CMC medium without any precursor addition. The de novo biosynthesis of caffeic acid from lignocellulose was achieved for the first time.

### Improving the production of caffeic acid by a multi-strain co-culture system

**Fig. 7:**
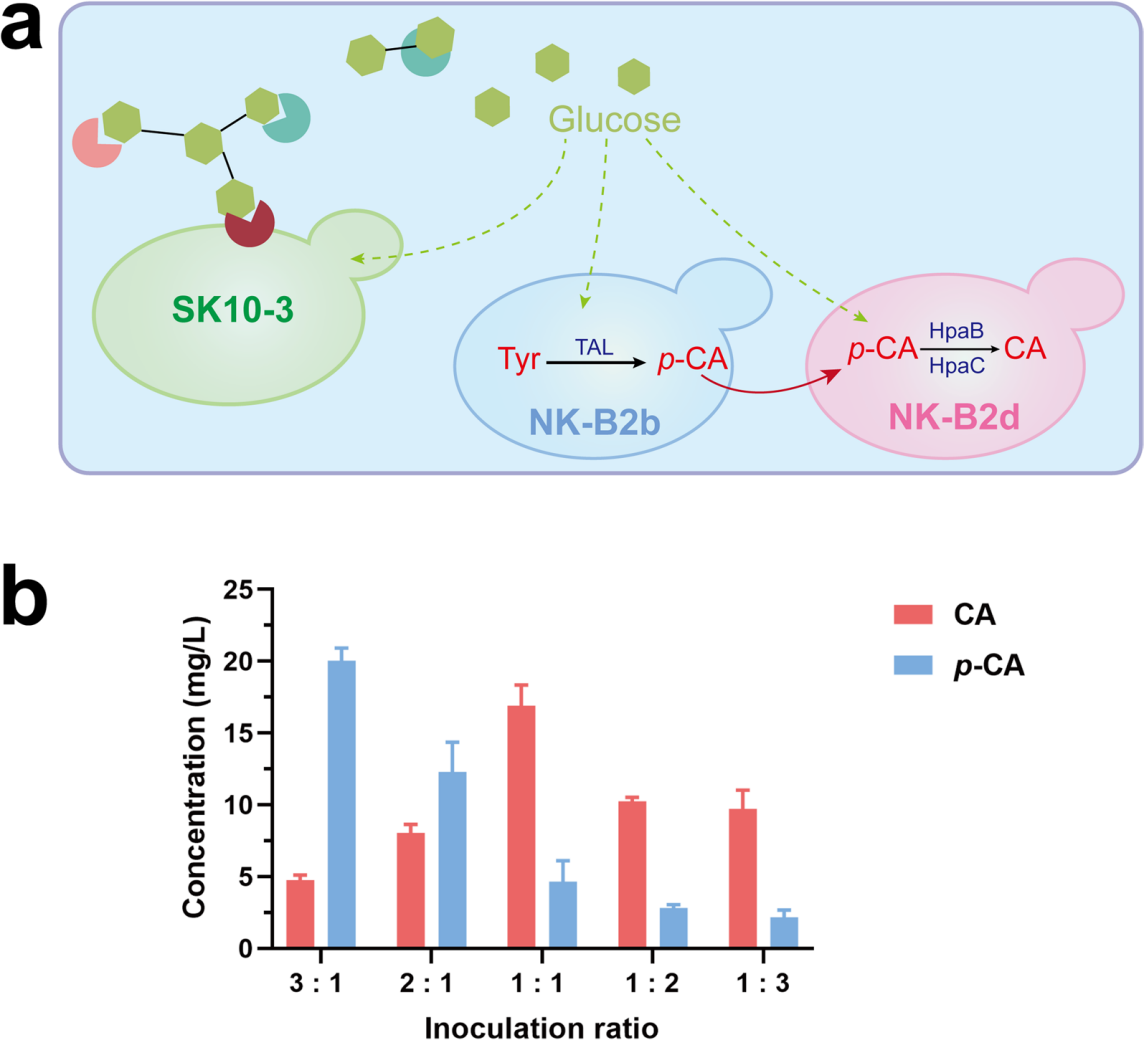
**a** Schematic illustration of the multi-strain co-culture system. **b** CA production and *p*-CA residue after 168 h fermentation in the multi-strain co-culture system with different inoculation ratios of NK-B2b to NK-B2d. Three replicates of each sample were used

It has been confirmed that caffeic acid synthases HpaB and HpaC are expressed in *S. cerevisiae* with high catalytic efficiency [37, 38]. However, in the co-culture system we studied, the titer of caffeic acid was only 8.33 mg/L, and there was a large amount of *p*-CA surplus (Fig. 6c). Accordingly, we speculated that under such low-sugar, unfavorable growth conditions, the caffeic acid biosynthesis pathway increased the growth pressure of the NK-B2c strain, thereby limiting the expression of *PahpaB* and *SehpaC*. The caffeic acid biosynthesis pathway was split into two strains, one is the NK-B2b strain, which expresses only the *RcTAL* gene, and the other is the NK-B2d strain, which expresses the *PahpaB* and *SehpaC* genes. A three-strain co-culture system was constructed to alleviate the metabolic pressure of the caffeic acid-producing strain (Fig. 7a). In this system, SK10-3 still accounted for one-third of the total biomass to meet the glucose supply. To find a balance between the other two strains to maximize caffeic acid production, different inoculation ratios of NK-B2b to NK-B2d (3:1, 2:1, 1:1, 1:2, and 1:3) were set, and the final caffeic acid titer was measured (Fig. 7b). After 168 h fermentation, 16.91 mg/L caffeic acid was accumulated, while NK-B2b and NK-B2d were inoculated equally, and the residual amount of *p*-CA was considerably reduced.

## Discussion

Mono-culture is the primary modality in microbial biosynthesis research. With the increasing complexity of biosynthesis pathways, mono-culture is extremely challenging. The advantages of co-culture strategies are gradually emerging, especially in the study of building complex and non-linear pathways. The co-culture strategy can flexibly modularize and divide an entire path to member strains, which is conducive to balance the biosynthesis ability between the modules. In the present study, we first achieved the bioconversion from lignocellulose to aromatic compounds, such as betaxanthin, *p*-CA, and caffeic acid. Although de novo synthesis from CMC to *p*-CA was also achieved in the pure culture system, SK10-3 possesses both the CMC degradation system and the *p*-CA synthesis pathway, which greatly increased its metabolic burden and resulted in extremely low final titers, not only in CMC medium but also in glucose medium. Li et al. emphasized the significance of pathway balancing; they improved the production of rosmarinic acid by modularizing and balancing the complex pathway into a three-strain co-culture system [39]. Our results are consistent with this finding; the production of *p*-CA in the final co-culture system increased by 155.9-fold as compared to that in mono-culture; thus, the de novo conversion of caffeic acid from CMC was achieved.

Presently, co-cultivation is an indispensable production mode in scientific research and industrial manufacturing. This strategy is highly significant as it enables the use of cheap substrates, improves the yield, and allows the development of new materials [40]. Previous studies have reported several applications of co-cultivation by using different species of microorganisms [41–45]. Several factors such as environmental conditions, nutrient types, and interaction relationships should be considered during the co-cultivation of strains of different species. The engineered *S. cerevisiae* strains were used in this research to avoid unknown interactions between strains of different species and to avoid negative feedback regulations that could inhibit strain growth and biosynthesis. *S. cerevisiae* has also been proved to be a robust organism that can be used for industrial-scale fermentation and has been widely used in the brewing industry [46] and pharmaceutical industry [47–49] as GRAS (generally recognized as safe). The high-efficiency utilization of lignocellulose as a carbon source has higher commercial value than high-cost food agriculture carbon sources.

Various factors also limit the synthesis of the products in this study. The first factor is the high polymerization of cellulose materials. A large amount of CMC was added to the medium to increase the content of carbon source; the medium was semi-solid, which limited the fluidity and CMC degradation efficiency of strains in the fermentation process. For this purpose, we attempted to replace other types of cellulosic materials and improve the cellulose-degrading performance of the strain. Second, in CMC medium, the biomass of strains was greatly restricted (Figs. 3 and 5), which directly affects the yield of final products. In the co-culture system, SK10-3 as a monosaccharides donor also consumes glucose, and only a part of carbon source can be absorbed by the fermentation strains. In future studies, SK10-3 will be engineered to metabolize other types of carbon sources, such as xylose; unnecessary metabolic pathways in member strains will also be eliminated to reduce metabolic energy consumption. Furthermore, fed-batch fermentation will be used to optimize the co-culture process.

## Conclusion

In this study, *p*-CA and caffeic acid were biosynthesized for the first time from lignocellulose, the most abundant renewable and non-food agricultural resource, through a co-culture strategy. After a series of optimization, *p*-CA production was increased to 71.71 mg/L, which is 155.9-fold higher than mono-culture. Caffeic acid with a higher application value was also de novo synthesized from CMC. Our findings demonstrate that the co-culture strategy has advantages in complex biosynthesis and is highly significant for industrial manufacturing, thus laying a foundation for the application of de novo biosynthesis of various high value-added products from lignocellulose.

## Materials and methods

### Strains, media, and mono-culture conditions

All strains and plasmids used in this study are listed in Table 2. Yeast strains were cultured at 30 °C with shaking at 220 rpm in SC medium [2% glucose, 0.5% (NH_4_)_2_SO_4_, 0.17% yeast nitrogen base without amino acids (YNB), and 0.13% amino acid mixture]. A drop-out synthetic medium without uracil or histone or uracil-histone (SC-ura or SC-his or SC-ura-his) was used to enrich strains carrying plasmids. These strains were then inoculated into 20 mL fresh SC medium or CMC medium [1% CMC, 0.5% (NH_4_)_2_SO_4_, 0.17% YNB, and 0.13% amino acid mixture] in 100 mL flasks with an optical density at OD_600_ of 0.1 and fermented continuously for 120 h.

**Table 2:** Strains and plasmids used in this study

Strains and plasmids	Relevant characteristics	Source
Strains		
BY4741	*MATα, ura3Δ0, leu2Δ0, his3Δ1, met15Δ0*	EUROSCARF, Frankfurt, Germany
BY4742	*MATa, ura3Δ0, leu2Δ0, his3Δ1, lys2Δ0*	EUROSCARF, Frankfurt, Germany
NK-B2	BY4742; *htz1Δ*	[22]
SK10-3	SK1 [BY4741 (*MATa, his3Δ1, leu2Δ0, met15Δ0, ura3Δ0*); *tpi1*:: *loxP*]; *δ*-integration of *BGLI*, *EGII*, *CBHI* genes	[36]
BY4741b	BY4741; pLC-c1	This study
SK10-3b	SK10-3; pLC-c1	This study
BY4742a	BY4742; pLC84	This study
BY4742b	BY4742; pLC-c1	[22]
NK-B2a	NK-B2; pLC84	[22]
NK-B2b	NK-B2; pLC-c1	[22]
NK-B2c	NK-B2b; pLC-c4	This study
NK-B2d	NK-B2; pLC-c4	This study
Plasmids		
pSP-G1	2 μm ori, *URA3*, P_*TEF1*_-T_*ADH1*_, P_*PGK1*_-T_*CYC1*_, Amp^r^	[50]
pLC84	pSP-G1:: P_*PGK1*_-Tyrosine hydroxylase-T_*CYC1*_, P_*TEF1*_-DOPA dioxygenase-T_*ADH1*_	[51]
pLC-c1	pSP-G1:: P_*PGK1*_-*RcTAL*-T_*CYC1*_, P_*TEF1*_-T_*ADH1*_	[23]
pLC41	2 μm ori, *HIS3*, P_*TEF1*_-T_*ADH1*_, P_*PGK1*_-T_*CYC1*_, Amp^r^	[52]
pLC-c4	pLC41:: P_*PGK1*_-*PahpaB*-T_*CYC1*_, P_*TEF1*_-*SehpaC*-T_*ADH1*_	This study

### Construction of strains and plasmids

The metabolically engineered strains SK10-3 and NK-B2 were constructed in our previous studies [22, 36]. Cellulose-degrading strains were constructed through the *POT1*-mediated *δ*-integration strategy [29] by integrating three codon-optimized cellulase genes encoding *Talaromyces emersonii* CBHI, *Trichoderma reesei* EGII, and *Aspergillus aculeatus* BGLI into yeast chromosomes. A series of recombinant strains with high cellulolytic activity were screened to degrade different cellulosic substrates [Avicel, CMC, and phosphoric acid swollen cellulose (PASC)] and produce bioethanol. SK10-3 is one of the engineered strains that can decompose CMC efficiently. The *htz1Δ* strain, NK-B2, was detected as a high-yielding strain of tyrosine, a precursor for various aromatic compounds.

The plasmid pLC-c4 was constructed in this study; the genes *PahpaB* and *SehpaC* were codon-optimized and synthesized by GENEWIZ (Suzhou, China). The Hieff Clone® Plus One Step Cloning Kit (Yeasen, Shanghai, China) was used in plasmid construction. The vector pLC41 was first linearized with *SmaI* and integrated with a *PahpaB* cassette. Subsequently, *SehpaC* was integrated into the *NotI* site of the plasmid by using the same method. The primers used are listed in Additional file 1: Table S3. The LiAc transformation method was used to introduce plasmids into yeast strains.

### Co-cultivation method

Before inoculation into the co-culture system, the strains were incubated in SC medium or drop-out medium for 24 h to prepare seed culture. The seed culture was centrifuged and washed, the cell pellets were then suspended in S solution [0.5% (NH_4_)_2_SO_4_, 0.17% YNB, and 0.13% amino acid mixture] and inoculated into CMC medium. In the experiment for verifying the effect of the inoculum ratio and interval time on *p*-CA production, the seed culture of SK10-3 was inoculated first and mono-cultured for 0, 12 or 24 h, and the NK-B2b seed culture was then inoculated with the total inoculum OD_600_ of 0.1 and fermented for another 120 h.

### HPLC analysis

The aromatic compounds in this study were quantified by an HPLC instrument (CoMetro 6000, NJ, USA) equipped with an ultraviolet detector (CoMetro 6000 PVW, NJ, USA) and a C18 column (250 mm × 4.6 mm, 5 μm, Agilent). *p*-CA and caffeic acid were detected at 310 nm wavelength (Fig. 6). A mixture of 5% acetonitrile and 0.1% trifluoroacetic acid in pure water was used as mobile phase A, while 0.1% trifluoroacetic acid in acetonitrile was used as mobile phase B. A sample volume of 10 μL was injected into the detector, and the flow rate was 1 mL/min. The samples were detected under a 35-min gradient program using the following conditions: 6% to 50% phase B for 13 min, 50% to 98% phase B for 13 min, 98% phase B for 3 min, 98% to 6% phase B for 12 min, and washing with 6% phase B for 4 min.

### Determination of glucose concentration

During the co-cultivation process, the glucose concentration in the medium was monitored by a glucose assay kit (Solarbio® BC2500). Samples were collected every 12 or 24 h, and the glucose concentration was then determined according to the manufacturer’s protocol. The readings were measured by a UV–vis spectrophotometer (Jinhua 752, Shanghai, China). Every experiment was performed at least three times.

### Determination of the ratio of the two *S. cerevisiae* strains after co-culture fermentation

A spotting plate experiment was performed to determine the ratio of SK10-3 and NK-B2b after co-culture fermentation. SK10-3 can grow on SC medium and CMC medium, while NK-B2b can survive only on SC medium. After co-culture fermentation, the yeast culture suspension was diluted and spread on a fresh SC medium plate. After 48 h of incubation, a count of 100 ~ 300 colonies per plate was considered to be appropriate. Next, 100 colonies were randomly selected and spotted on a fresh CMC medium plate. Finally, the number of surviving colonies on the CMC plates was counted; these colonies belonged to the SK10-3 strain, and the remaining colonies that did not grow on the CMC plates were of the NK-B2b strain. The ratio of these two strains was then accordingly calculated. Every experiment was performed at least three times.

## Supplementary Information

**Additional file 1:** **Table S1.** Glucose content during the co-culture of SK10-3 and NK-B2b in 10 g/L CMC medium with different inoculum ratios and interval times. **Table S2.** Glucose content during the co-culture of SK10-3 and NK-B2b in rich CMC medium with different inoculum ratios and interval times. **Table S3.** Primers for plasmids construction

## Data Availability

All the data and materials supporting the findings of this article are included within the article and its additional files.
