# Supplementary material for: De novo biosynthesis of p-coumaric acid and caffeic acid from carboxymethyl-cellulose by microbial co-culture strategy
Source: Microb Cell Fact. 2022 May 10;21:81. doi: 10.1186/s12934-022-01805-5 (PMC9088102; doi:10.1186/s12934-022-01805-5)
Supplement: Supplementary file 1 — Additional file 1: Table S1. Glucose content during the co-culture of SK10-3 and NK-B2b in 10 g/L CMC medium with different inoculum ratios and interval times. Table S2. Glucose content during the co-culture of SK10-3 and NK-B2b in rich CMC medium with different inoculum ratios and interval times. Table S3. Primers for plasmids construction [file 12934_2022_1805_MOESM1_ESM.docx]

***De novo* biosynthesis of *p*-coumaric acid and caffeic acid from** **carboxymethyl-cellulose by microbial co-culture strategy**

Miao Cai ^1^, Jiayu Liu ^1^, Xiaofei Song ^2^, Hang Qi ^1^, Yuanzi Li ^3^, Zhenzhou Wu ^1^, Haijin Xu ^1,^*, Mingqiang Qiao ^1,^*

^1^ The Key Laboratory of Molecular Microbiology and Technology, Ministry of Education, College of Life Sciences, Nankai University, Tianjin 300071, China

^2^ College Biotechnology and Bioengineering, Zhejiang University of Technology, Hangzhou 310014, China

^3^ School of Light Industry, Beijing Technology and Business University (BTBU), Beijing 100048, China

*Corresponding authors: E-mail: [nkxuhaijin@163.com](mailto:nkxuhaijin@163.com); [qiaomq@nankai.edu.cn](mailto:qiaomq@nankai.edu.cn)

**Table S1.** Glucose content during the co-culture of SK10-3 and NK-B2b in 10 g/L CMC medium with different inoculum ratios and interval times

| **The interval time** | **The ratio of SK10-3 to NK-B2b** | **Glucose content (mg/L) during co-cultivation** | | | | |
| --- | --- | --- | --- | --- | --- | --- |
|  |  | **12 h** | **24 h** | **36 h** | **48 h** | **60 h** |
| 0 h | 3 : 1 | 58.86±5.04 | 9.72±1.18 | 1.14±0.63 | 4.20±0.63 | 2.10±0.68 |
|  | 2 : 1 | 40.80±3.57 | 73.92±3.87 | 3.54±1.50 | 7.74±0.72 | 0.42±0.42 |
|  | 1 : 1 | 26.40±2.18 | 79.38±2.52 | 7.74±0.65 | 6.66±0.65 | 1.86±0.55 |
|  | 1 : 2 | 15.18±1.70 | 48.36±5.68 | 27.72±0.95 | 12.24±1.12 | 3.96±0.54 |
|  | 1 : 3 | 9.00±2.16 | 21.84±2.62 | 40.80±10.85 | 30.96±1.08 | 5.88±0.99 |
| 12 h | 3 : 1 | 80.82±5.19 | 14.58±0.72 | 0.24±0.27 | - | - |
|  | 2 : 1 | 59.58±5.75 | 15.12±0.82 | 0.72±0.36 | - | - |
|  | 1 : 1 | 56.94±2.52 | 28.80±4.07 | 1.86±0.27 | - | - |
|  | 1 : 2 | 80.58±4.99 | 27.48±1.53 | 0.90±0.31 | - | - |
|  | 1 : 3 | 62.16±5.86 | 22.62±1.63 | 1.14±0.63 | - | - |
| 24 h | 3 : 1 | 17.52±1.53 | 7.20±1.60 | - | - | - |
|  | 2 : 1 | 19.68±2.93 | 8.70±1.50 | - | - | - |
|  | 1 : 1 | 24.60±4.17 | 6.96±1.63 | - | - | - |
|  | 1 : 2 | 42.84±4.66 | 5.88±1.40 | - | - | - |
|  | 1 : 3 | 72.30±2.31 | 12.66±1.17 | - | - | - |

-, indicates no glucose was detected

**Table S2.** Glucose content during the co-culture of SK10-3 and NK-B2b in rich CMC medium with different inoculum ratios and interval times

| **The final content of CMC (g/L)** | **The ratio of SK10-3 to NK-B2b** | **Glucose content (mg/L) during co-cultivation** | | | | | | |
| --- | --- | --- | --- | --- | --- | --- | --- | --- |
|  |  | **24 h** | **48 h** | **72 h** | **96 h** | **120 h** | **144 h** | **168 h** |
| 20 | 1 : 1  (0 h interval) | 12.18±0.91 | 18.72±1.95 | 9.12±0.58 | 2.34±0.72 | 74.46±1.67 | 40.08±1.92 | 14.64±0.81 |
|  | 1 : 2  (0 h interval) | 15.78±0.68 | 20.94±1.36 | 16.38±2.00 | 9.54±1.08 | 95.94±8.61 | 43.80±0.68 | 12.48±0.63 |
|  | 1 : 3  (12 h interval) | 7.20±1.60 | 13.56±1.05 | 12.42±1.57 | 9.90±0.95 | 94.92±2.25 | 64.14±1.66 | 13.98±1.33 |
| 30 | 1 : 1  (0 h interval) | 4.80±0.73 | 9.36±0.65 | 11.34±1.43 | 15.18±0.85 | 168.06±2.34 | 129.54±2.79 | 31.92±1.55 |
|  | 1 : 2  (0 h interval) | 12.60±1.60 | 19.44±1.12 | 15.54±1.98 | 21.96±2.42 | 85.74±2.97 | 49.50±2.25 | 8.64±1.26 |
|  | 1 : 3  (12 h interval) | 11.16±0.78 | 16.98±1.45 | 17.16±1.97 | 14.94±1.60 | 139.02±2.40 | 98.70±2.65 | 22.68±1.00 |

**Table S3.** Primers for plasmids construction

| **Primers** | **Sequence (5’-3’)** | **Applications** |
| --- | --- | --- |
| F1 | CGACTCACTATAGGGCCCGGGATGAAACCTGAAGATTTTAGAGCTTCT | Amplify *PahpaB* gene for pLC-c4 construction, forward primer |
| R1 | TGTTCCATGTCGACGCCCGGGTTATTGTCTAATTCTATCCAAAACATTAATATC | Amplify *PahpaB* gene for pLC-c4 construction, reverse primer |
| F2 | AAGTTTTAATTACAAGCGGCCGCATGCAAGTTGATGAACAAAGATTGA | Amplify *PahpaC* gene for pLC-c4 construction, forward primer |
| R2 | TCCATCGATACTAGTGCGGCCGCTTAAACAGGAGCTTCCATTTCCA | Amplify *PahpaC* gene for pLC-c4 construction, reverse primer |

Overlaps are underlined.
